# TRPM2: bridging calcium and ROS signaling pathways—implications for human diseases

**DOI:** 10.3389/fphys.2023.1217828

**Published:** 2023-07-27

**Authors:** Maria Maliougina, Yassine El Hiani

**Affiliations:** Department of Physiology and Biophysics, Dalhousie University Faculty of Medicine, Halifax, NS, Canada

**Keywords:** TRPM2 cation channels, oxidative stress, calcium signal, bioenergetics, oxidant defense, autophagy

## Abstract

TRPM2 is a versatile and essential signaling molecule that plays diverse roles in Ca^2+^ homeostasis and oxidative stress signaling, with implications in various diseases. Research evidence has shown that TRPM2 is a promising therapeutic target. However, the decision of whether to activate or inhibit TRPM2 function depends on the context and specific disease. A deeper understanding of the molecular mechanisms governing TRPM2 activation and regulation could pave the way for the development of innovative therapeutics targeting TRPM2 to treat a broad range of diseases. In this review, we examine the structural and biophysical details of TRPM2, its involvement in neurological and cardiovascular diseases, and its role in inflammation and immune system function. In addition, we provide a comprehensive overview of the current knowledge of TRPM2 signaling pathways in cancer, including its functions in bioenergetics, oxidant defense, autophagy, and response to anticancer drugs.

## Highlights

• TRPM2 converts ROS signal into a Ca^2+^ signal.
• TRPM2 is involved is various disease.
• Both activation and inhibition of TRPM2 could be used in therapy.

## Introduction

Transient Receptor Potential (TRP) channels are a large superfamily of signaling membrane proteins. They are widely expressed and have similar overall structure, yet they possess distinct regulatory mechanisms triggered by an astonishing variety of stimuli, ranging from ions and oxidants to photons and mechanical force. In humans, 27 TRP genes have been described and classified into six subfamilies based on sequence homology: TRPA (ankyrin), TRPC (canonical, seven members), TRPM (melastatin, eight members), TRPML (mucolipin, three members), TRPP (polycystin, two members), and TRPV (vanilloid, six members) (Moran et al., 2011; Himmel and Cox, 2020; Fallah et al., 2022).

TRPM2 is ubiquitously expressed and naturally activated by adenosine ribose (ADPR), a molecule produced in the nucleus and mitochondria under oxidative stress conditions (Perraud et al., 2001; Malko and Jiang, 2020). In the nucleus, excessive accumulation of reactive oxygen species (ROS) leads to DNA damage, which triggers the production of poly-ADPR polymerase (PARP) and poly-ADPR glycohydrolase (PARG). These enzymes catalyze the conversion of nicotinamide dinucleotide (NAD^+^) to ADPR (Fonfria et al., 2004; Miller, 2004). In mitochondria, NUDT9-ADPRase converts NAD^+^ to ADPR (Perraud et al., 2005). Once formed, ADPR diffuses and binds to the cytoplasmic portion of TRPM2 at the plasma membrane, activating calcium (Ca^2+^) influx. This ability of TRPM2 to sense and respond to oxidative stress signals by converting the ROS signal to a Ca^2+^ signal places TRPM2 at the heart of the cellular signaling network, which buffers oxidative stress and regulates Ca^2+^ homeostasis—two major signaling pathways important for all cellular functions.

Despite two decades of elaborate studies and research investigations, many aspects of TRPM2 function and regulation have only recently been elucidated, and many more questions remain unanswered. In this review, we provide an overview of the functional structure of the TRPM2 channel, its mode of activation and gating mechanism. We review the roles of TRPM2 in various neurological and cardiovascular diseases as well as in inflammation and the immune system. Lastly, we examine the current understanding of TRPM2-mediated cancer bioenergetics, oxidant defense, autophagy and response to anticancer drugs.

## TRPM2—structure and gating mechanism

**FIGURE 1:**
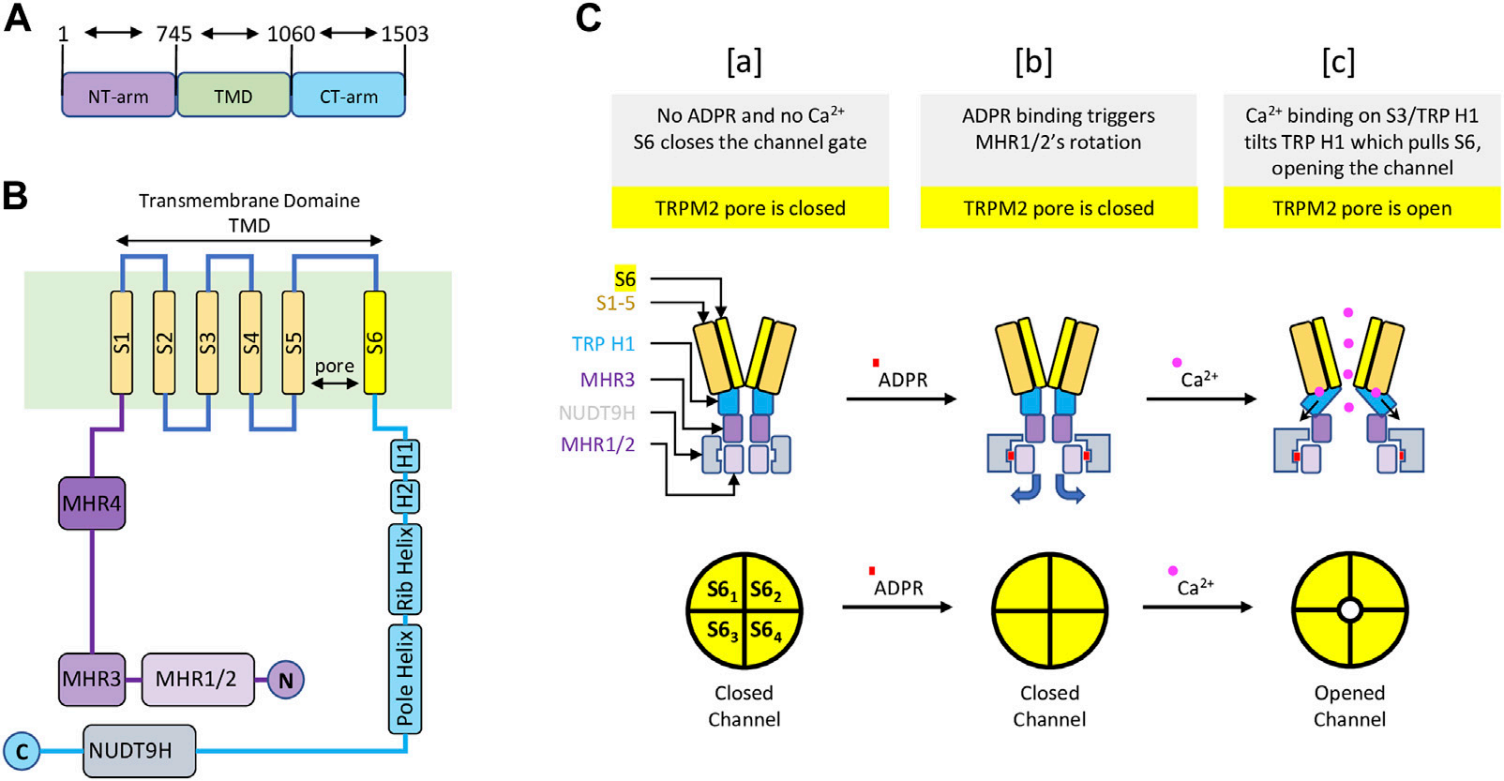
Schematic representation of the main structure of the TRPM2 channel and the gating mechanism—Modified from (Wang et al., 2018) **(A)** TRPM2 major domain sizes, with residue numbers indicating the start and end of each domain. **(B)** TRPM2 special organization showing 1) the transmembrane domain composed of six transmembrane segments (TMS) with a pore between fifth and sixth TMS; 2) a cytoplasmic TRPM2 N-terminal containing four homology regions (MHR1-4); 3) a cytoplasmic TRPM2 C-terminal displaying the NUDT9H domain, where ADPR initiate TRPM2 activation. **(C)** A diagram representing TRPM2 gating. [a] In the absence of ADPR and Ca^2+^, transmembrane segment 6 (S6) (yellow) closes the channel gate of TRPM2 (top left). In this condition, the pore of the TRPM2 homotetramer is closed (lower left). [b] Upon ADPR binding, NUDT9H (grey) undergoes conformational changes that trigger rotation of MHR1/2 (thin purple), exposing the channel vestibule (top middle). In this condition, the pore of TRPM2 homotetramer is closed (bottom center). [c] ADPR-mediated conformational changes allow Ca^2+^ binding to TMS3 (orange) and TRP H1 (cyan), causing TRP H1 to tilt and pull S6, resulting in rotation of S6 and channel opening (top right). In this state, the pore of the TRPM2 homoteramer is open (bottom right).

TRPM2 is the second member of the family of TRPM channels, which is part of the larger TRP channel superfamily. Like other TRP channels, TRPM2 has a modular architecture consisting of six transmembrane segments (TMS)s, with a pore between the fifth and sixth TMS, and cytoplasmic N and C termini. Like other TRPM members, TRPM2 exhibits similar features such N-terminal homology regions (MHR1-4) and C-terminal domains (TRP helices H1 & H2, Rib helix and Pole helix). However, unlike all other TRP channel members, TRPM2 possesses a unique NUDT9-H domain at its C-terminus that senses ADPR and shares similarities with the mitochondrial NUDT9 enzyme, which cleaves ADPR into adenosine monophosphate (AMP) and ribose 5-phosphate (R5P) (Clapham, 2003; Perraud et al., 2003) (Figure 1). Whether NUDT9-H of TRPM2 also cleaves ADPR into AMP and R5B like the mitochondrial NUDT9 enzyme remains controversial. Nevertheless, Csanàdy’s group has provided evidence suggesting that the NUDT9H domain of TRPM2 does not function as an ADPR hydrolase, and that TRPM2 is a simple ligand-gated channel rather than a ‘chanzyme’ (Toth et al., 2014; Iordanov et al., 2016).

The functional TRPM2 channel exists as a homotetramer that forms a Ca^2+^ permeable, cationic, nonselective pore with a unique gating mechanism (Wang et al., 2018; Zhang et al., 2018). The gating mechanism of human TRPM2 involves the sequential binding of ADPR and Ca^2+^. In the closed state—without ADPR and Ca^2+^—the NUDT9-H region interacts closely with the MRH1/2 and MRH3 domains of the same subunit and the MRH1/2 region of the adjacent subunit within the homotetramer. The binding of ADPR to NUDT9-H triggers structural changes, resulting in a dramatic 27° counterclockwise rotation of the NUDT9-H and MHR1/2 regions, which releases the vestibule space of the channel pore. At this point, the ADPR-bound TRPM2 is not open but is primed for channel opening by Ca^2+^ binding. The conformational changes mediated by ADPR expose the TMS environment, allowing for further structural changes upon Ca^2+^ binding (Wang et al., 2018). Ca^2+^ binding occurs at four sites: E843 and Q846 of TMS2, N869 of TMS3, and E1073 of the TRP H1 helix (Csanady and Torocsik, 2009; Wang et al., 2018). Following Ca^2+^ binding, the TRP H1 helix undergoes a conformational change and interacts to pull TMS6, thereby widening the channel pore and opening the channel gate. Cytosolic Ca^2+^ is thought to initiate the opening of the channel, only to remain activated by new extracellular Ca^2+^ entering the pore domain. Altogether, the coactivation of TRPM2 by ADPR and Ca^2+^ is controlled by the C-terminal NUDT9-H and TMS domains (Wang et al., 2018). In its unbound state, NUDT9-H forms intra- and inter-subunit interactions to lock the pore vestibule and, consequently, the channel gate. The binding of ADPR to NUDT9-H unlocks the channel vestibule, and Ca^2+^ binding opens the channel gate (Figure 1).

Upon activation, the whole-cell TRPM2 current exhibits a stable and linear current-voltage (I/V) relationship, reversing at 0 mV (Perraud et al., 2001; Sano et al., 2001). In excised patches, TRPM2 unitary currents also display a linear I/V shape with a conductance range of 52–60 pS at negative potentials and ∼72 pS at positive potentials, and a reversal potential around 0 mV, suggesting that TRPM2 functions as a non-selective cation channel (Perraud et al., 2001; Sano et al., 2001). Indeed, extensive research has demonstrated that TRPM2 is permeable to various monovalent and divalent ions, including sodium (Na^+^) potassium (K^+^), Ca^2+^, and magnesium (Mg^2+^). The permeability ratios were found to 1.1 for the ratio of K^+^ to Na^+^ (P_K+_/P_Na+_), 0.9 for the ratio of Ca^2+^ to Na^+^ (P_Ca2+_/P_Na+_), and 0.5 for the ratio of Mg^2+^ to Na^+^ (P_Mg2+_/P_Na+_) (Perraud et al., 2001; Sano et al., 2001; Kraft et al., 2004; Xia et al., 2008). In this context, Xia and coworkers demonstrated that side-chain residues G960, A987, and G981 control permeation of TRPM2 for Ca2+, and a fourth side-chain residue G1022 contributes to Mg^2+^ permeation, all of which lie within the selectivity filter of the TRPM2 pore (Xia et al., 2008).

In contrast to whole-cell TRPM2 currents, which remain stable as long as both ADPR and Ca^2+^ are present (Perraud et al., 2001; Sano et al., 2001), inside-out TRPM2 currents rapidly decay within 1–2 min (Sano et al., 2001; Hill et al., 2006; Csanady and Torocsik, 2009). This decay continues until it is completely suppressed, even in the continued presence of saturating concentrations of ADPR and Ca^2+^ in excised patches. This so-called rundown poses substantial challenges to obtaining steady-state single-channel recordings, thereby limiting the proper investigation of the biophysical details of TRPM2. Csanáday’s group has extensively investigated the mechanism behind this decay in TRPM2 and demonstrated that TRPM2 “rundown” is due to a conformational rearrangement of the selectivity filter, causing a progressive, irreversible decline in the number of activated channels (Csanady and Torocsik, 2009). Indeed, the introduction of one negatively charged side chain into Y985E significantly slowed the decay’s time-course of TRPM2 current and prevented its complete inactivation (Toth and Csanady, 2012). Consequently, a significant portion of the TRPM2 current remained constant for as long as the patches could be held. This mechanism is similar to those described in TRPM4 and TRPM5, where the removal of one or two side chain residues induced a rapid, irreversible rundown (Liu and Liman, 2003; Zhang et al., 2005; Nilius et al., 2006; Liman et al., 2007). Indeed, when the researchers introduced two negative charges plus a single-residue insertion in TRPM2, mimicking the TRPM5 selectivity filter, the result was non-inactivating TRPM2 channels with stable, unabated maximal currents after activation (Toth and Csanady, 2012). While this non-inactivating TRPM2 variant displayed altered permeation properties, it has no impact on the channel pore diameter and an intact ADPR/Ca2+-dependent gating. This opens the door for comprehensive studies of the biophysical details of TRPM2 gating, as well as the identification of more selective, specific, and potent modulators of TRPM2 channel.

## TRPM2—the master of CA^2+^ and ROS signaling interplay

If Ca^2+^ and ROS represent the two most vital second messengers used by a cell in response to both intra- and extracellular signals, their interplay forms a critical cell network involved in virtually every aspect of cellular physiology (Yan et al., 2006; Hidalgo and Donoso, 2008; Gorlach et al., 2015). On the one hand, Ca^2+^ signals modulate the production of ROS by stimulating the Krebs cycle and oxidative phosphorylation in mitochondria (Brookes et al., 2004), and by regulating NADPH oxidases at the endoplasmic and plasma membranes (Jiang and Zhang, 2003; Kobayashi et al., 2007; Salmon and Ahluwalia, 2009). On the other hand, ROS signals regulate the activity of several Ca^2+^ channels and transporters (Poteser et al., 2006; Takahashi and Mori, 2011; Bogeski et al., 2012; Nikolaienko et al., 2018; Schmidt et al., 2019). Therefore, understanding the reciprocal regulation of ROS and Ca^2+^ is of paramount importance for preventing dysfunctions that lead to pathological conditions.

In this context, particular attention has been given to the Ca^2+^ permeable TRPM2 channel due to its regulation by oxidative stress (Hara et al., 2002; Fonfria et al., 2004; Perraud et al., 2005), and its emerging role in diverse physiological processes. One of its most critical functions lies within the immune system, where it mediates the oxidative burst in neutrophils and plays a role in inflammatory responses (Knowles et al., 2013). Additionally, TRPM2 has been found to be involved in insulin secretion from pancreatic β cells (Togashi et al., 2006; Uchida et al., 2011; Uchida and Tominaga, 2011), pain (Haraguchi et al., 2012; So et al., 2015; Matsumoto et al., 2016), and regulating body temperature (Song et al., 2016; Kashio and Tominaga, 2017; Tan and McNaughton, 2018; Vilar et al., 2020). At the cellular level, the activation of the TRPM2 channel, typically through oxidative stress, allows for an influx of Ca^2+^ ions into the cell, altering the intracellular Ca^2+^ concentration. This change in Ca^2+^ homeostasis can subsequently influence various intracellular processes, including cell signaling, gene expression, cell proliferation, and apoptosis.

This critical position at the interface of the Ca^2+^ and ROS systems highlights the crucial role of TRPM2 in fine-tuning cellular signaling and, ultimately, determining a cell’s fate (Yamamoto and Shimizu, 2016). However, this also implies that any disturbance in TRPM2 expression and/or regulation can affect the Ca^2+^ and/or ROS systems and potentially cause various pathophysiological disorders. Indeed, dysfunction or dysregulation of TRPM2 has been linked with a range of pathological conditions, including neurodegenerative diseases, cardiovascular diseases, and cancer, thus underscoring its pivotal role in maintaining cellular health and function.

### TRPM2 in aging and neurodegenerative disease

Longstanding evidence have demonstrated a strong correlation between altered ROS and Ca^2+^ systems and brain diseases, including aging and neurodegenerative disorders (Patel, 2016; Cobley et al., 2018; Franco and Vargas, 2018; Madreiter-Sokolowski et al., 2020). Given its role in sensing and responding to oxidative stress, many research groups investigated the potential role of TRPM2 in oxidative stress-mediated brain diseases. One of the most markers of aging is probably the dramatic decline of the antioxidant Glutathione (GSH). In this context, Belrose and coworkers showed that the loss of GSH-mediated neuronal senescence is associated with increased TRPM2 current density in cultured hippocampal neurons, suggesting a cellular connexion between activation of TRPM2 and neuronal senescence (Belrose et al., 2012). Furthermore, Kaneko’s group demonstrated that knocking out the TRPM2 channel attenuates cognitive dysfunction and prevents age-related damage in the white matter and hippocampus of normal and chronic cerebral hypoperfusion mouse models, suggesting that activation of TRPM2 has a deleterious effect on the pathophysiology of the aging brain (Miyanohara et al., 2018; Kakae et al., 2019). Activation of TRPM2 has also been associated with the establishment of long-term depression (LTD), a specific form of NMDAR-dependent synaptic plasticity that requires endocytosis of AMPA receptors (AMPAR) (Xie et al., 2011). Mechanistically, the authors showed that loss of TRPM2 impairs NMDAR-dependent LTD through the inactivation of GSK-3β and reduction of PSD95 and AMPAR Glu1 expression (Xie et al., 2011). The genetic etiology of TRPM2 has also been associated with increased susceptibility to bipolar disorder (BD). Indeed, a family-based association study showed the association between D543E mutation of Trpm2 and type-1 BD (McQuillin et al., 2006; Xu et al., 2009). Using TRPM2 KO mice, Jong and colleagues demonstrated that loss of TRPM2 is associated with the behavioral manifestations of BD, including increased anxiety and impaired social interaction (Jang et al., 2015). Mechanistically, the authors provided evidence that loss of TRPM2 induces the inhibitory phosphorylation of GSK-3 via calcineurin, a known kinases in bipolar disorder (Li et al., 2010; Pandey et al., 2010), and suggest that loss of TRPM2 has a deleterious effects on BD (Jang et al., 2015).

TRPM2 has also been associated with Alzheimer’s disease (AD), a common neurodegenerative disorder primarily associated with the accumulation of β-amyloid (Aβ) plaques, Ca^2+^ overload, and increased ROS (Gibson, 2002; Leclerc et al., 2009; Zhao and Zhao, 2013; Tong et al., 2018). For instance, inhibition of TRPM2 has been shown to reduce Aβ-mediated cytosolic Ca^2+^ overload and cytotoxicity in rat striatal cells, suggesting a key role of TRPM2 in Aβ-induced cytotoxicity (Fonfria et al., 2005). In support, another study also demonstrated that TRPM2-mediated massive Ca^2+^ entry is required for Aβ-mediated cerebrovascular dysfunction, confirming the role of the activation of TRPM2 in the deleterious cerebrovascular effects of Aβ (Park et al., 2014). Mechanistically, the authors showed that Aβ-mediated oxidative–nitrosative stress triggers PARP activity, DNA damage, and ADPR overproduction, which in turn activates TRPM2-mediated Ca^2+^ overload in cerebrovascular cells, ultimately impairing cerebral blood flow (Park et al., 2014).

Another hallmark of AD is microglia’s failure to phagocyte Aβ (Chen and Trapp, 2016; Sarlus and Heneka, 2017), due to the failure of Aβ-mediated NLRP3 activation and cognitive dysfunction (Halle et al., 2008; Gold and El Khoury, 2015). In this context, Amizadeh and coworkers demonstrated that the DPQ TRPM2 inhibitor decreases Aβ-mediated ROS and Ca^2+^ elevation, together with decreased NLRP3 activation and proinflammatory cytokines IL-1β secretion in microglial cells (Aminzadeh et al., 2018). Meanwhile, Ostapchenko and collaborators showed that Aβ-mediated decreases in the presynaptic marker synaptophysin and reduced microglial activation critically depend on TRPM2 current activation in cultured neurons. Using a Barnes maze analysis, the authors further show that inhibition of TRPM2 prevents the pathological and behavioral deficits observed in older (12–15 months) APP/PS1 AD mouse models, supporting a causative relationship between Aβ and the TRPM2 activation in Aβ-induced neurotoxicity and cognitive dysfunction (Ostapchenko et al., 2015). Interestingly, Li and Jiang showed that Aβ-mediated primary hippocampal neuron toxicity involves TRPM2-mediated lysosomal Zn^2+^ release (Li and Jiang, 2018). The authors demonstrated that Aβ-mediated neuron toxicity is ablated in TRPM2 KO mice and provided evidence that lysosomal TRPM2-mediated Zn^2+^ release causes lysosomal dysfunction and cytoplasmic Zn^2+^ overload, which in leads to mitochondrial Zn^2+^ accumulation and loss of mitochondrial function (Li and Jiang, 2018). Collectively, these results highlight the therapeutic potential of TRPM2 and suggest the inhibition of TRPM2 as promising strategy to counteract the deleterious effects of in brain diseases.

### TRPM2 in ischemic stroke

Ischemic stroke is characterized by sudden neurological deficits and occurs when the arteries supplying blood to the brain become blocked, resulting in neuronal necrosis (Dirnagl et al., 1999; Paul and Candelario-Jalil, 2021). A crucial mechanism involved in ischemic stroke is the excessive release of the neurotransmitter glutamate, which leads to overactivation of NMDA (N-methyl-D-aspartate) receptors (NMDAR) and subsequently to excessive Ca^2+^ influx and overproduction of ROS in neurons (Wu and Tymianski, 2018; Ge et al., 2020). Given the potential link between the Ca^2+^/ROS system and TRPM2 on the one hand and NMDAR-mediated ischemic stroke on the other, the role of TRPM2 in ischemic stroke has been investigated in detail (Turlova et al., 2018; Zong et al., 2022a; Xu et al., 2022). The first evidence linking TRPM2 with ischemic stroke was provided by Fonfria and collaborators, who showed increased TRPM2 mRNA expression and function after ischemic injury in the microglia of the transient middle cerebral artery occlusion (tMCAO) rat stroke model. They suggested that TRPM2 activation during ischemic brain injury may mediate key aspects of microglial pathophysiological responses (Fonfria et al., 2006). A similar result was also observed in neonatal mice, where genetic deletion of TRPM2 reduced brain injury and improved functional behavior after neonatal hypoxic-ischemic brain injury, suggesting TRPM2 inhibition as a promising therapeutic target for the ischemic brain injury (Huang et al., 2017). Intriguingly, the therapeutic benefit of TRPM2 inhibition in ischemic cell death appears sex-dependent (Yuan et al., 2009; Liu et al., 2011). Indeed, Jia and coworkers demonstrated that TRPM2-mediated ischemic cell death is male-specific and does not contribute to damage in female neurons or brain tissue. Using pharmacological and genetic detection of TRPM2, the authors demonstrated significant protection only for male neurons following *in vitro* ischemia (oxygen–glucose deprivation, OGD), while having no effect on female neurons (Jia et al., 2011). One explanation for this sex-dependent TRPM2 effect in stroke cell death may reside in a greater sensitivity to ADPR in males and/or activation of different signaling pathways in males *versus* females after ischemic injury (Lang and McCullough, 2008). Indeed, deletion of PARP, a potent signal for TRPM2 activation, has been shown to be neuroprotective in males and deleterious in females (McCullough et al., 2005), where stroke-mediated cell death involves caspase-dependent apoptosis (Renolleau et al., 2007; Liu et al., 2011).

​Mechanistically, TRPM2-mediated ischemic stroke has been associated with the expression and signalling of NMDARs (Ge et al., 2020; Xu et al., 2022). Among the numerous subunits, GluN2A and GluN2B are the most important types of NMDARs and have been shown to control signalling pathways leading to cell survival and death, respectively (Sun et al., 2018). Using an *in vivo* stroke model, Alim and coworkers demonstrated a shift in the GluN2A/GluN2B ratio toward the induction of GluN2A subunits and activation of the downstream prosurvival signalling pathways Akt and ERK1/2 in TRPM2 KO mice (Alim et al., 2013). A recent study further revealed that TRPM2 directly interacts with GluN2A to modulate its membrane expression (Boyce and Thompson, 2022; Zong et al., 2022b). The interaction between TRPM2 and GluN2A occurs via the unique EE3 motif at the N-terminus of TRPM2 and the KKR motifs at the C-terminus of GluN2A, requiring the recruitment of PKCγ to selectively increase NMDAR subunit activity and membrane expression (Zong et al., 2022b). Utilizing the TRPM2-derived interfering peptide TAT-EE3, the authors demonstrated that disruption of the TRPM2-NMDAR interaction effectively prevented phosphorylated CREB and ERK1/2 levels, inhibited neuronal excitotoxicity, and protected mice from ischemic stroke *in vitro* and *in vivo* (Zong et al., 2022b). This confirms the neuroprotective role of TRPM2 inhibition in stroke and suggests that peptide-based uncoupling of TRPM2-NMDAR association is a promising therapeutic strategy for ischemic stroke (Wu and Tymianski, 2018; Zong et al., 2022b). Overall, inhibition of TRPM2 could be an effective therapeutic target for the treatment of stroke in men.

### TRPM2 in cardiac ischemic/reperfusion

Myocardial infarctions are caused by sudden blood obstruction, leading to cardiac ischemia, which results from a decreased oxygen and glucose supply (Heusch, 2016; Heusch, 2019). After the ischemic episode, effective blood flow is restored, and the ischemic tissue is reperfused. However, this process leads to a secondary effect called ischemia/reperfusion (I/R) injury, characterized by ROS and Ca^2+^ overload, excessive production of inflammatory cytokines, and ultimately cardiomyocyte cell death (Hausenloy et al., 2017; Wu et al., 2018). Given the role of TRPM2 in the ROS/Ca^2+^ system, many research groups investigated the role of TRPM2 in the pathophysiology of I/R injury (Zhan et al., 2016). Yang and coworkers provided the first evidence that TRPM2 channels are functionally present in the plasmalemma of myocytes and are involved in ROS-mediated cardiomyocyte death. They showed that increased ROS triggers PAPR-induced ADPR production, which subsequently activates TRPM2, leading to mitochondrial Ca^2+^/Na^+^ uptake and accumulation. This process disrupts mitochondrial permeability, causing cytochrome C release and caspase 3 activation, ultimately triggering apoptotic cell death (Yang et al., 2006). Similarly, TRPM2 has also been associated with TNF-ɑ-mediated cardiomyocyte death (Roberge et al., 2014), an important proinflammatory cytokine involved in I/R (Kleinbongard et al., 2011). The authors showed that TNF-ɑ activates PARP and TRPM2, resulting in a massive Ca^2+^ influx that likely leads to mitochondrial Ca^2+^ overload. This, in turn, results in increased mitochondrial ROS production, caspase 8 activation, and ultimately cardiomyocyte death (Roberge et al., 2014). Collectively, these findings emphasize the significance of PARP and TRPM2 in I/R-mediated cardiomyocyte apoptosis and propose TRPM2 as a promising approach to mitigate I/R-mediated cardiovascular damage. However, Miller’s group conducted a series of studies that meticulously demonstrated that TRPM2 may actually play a protective role in I/R-mediated cardiovascular damage (Miller et al., 2013; Miller et al., 2014; Hoffman et al., 2015). They employed a cardiac ischemia period followed by reperfusion in WT and TRPM2-KO mice. They discovered that TRPM2-KO mice exhibit altered expression of proteins involved in contractility (higher Na^+^-Ca^2+^ exchanger, decreased α1-subunit of Na^+^-K^+^-ATPase), ROS scavenging (lower SOC1/2 and increased NOX1), and mitochondrial bioenergetics and mitophagy (lower complexes I, III, and IV, and increased complexes II and V; lower MCU and BNIP3). Consequently, TRPM2-KO mice have reduced mitochondrial function, including lower oxygen consumption rate, decreased ATP production, and increased mitochondrial ROS production. Importantly, rescue experiments with ectopic TRPM2 injections in TRPM2-KO mice confirmed that only TRPM2-WT, and not TRPM2-E960D silent pore, can rescue and restore myocyte mitochondrial function, corroborating the protective role of TRPM2-mediated Ca^2+^ entry in cardiomyocyte survival after I/R. In summary, these results suggest that TRPM2-mediated Ca^2+^ influx into myocytes serves a dual protective function against I/R injury by maintaining mitochondrial function and energy production and by promoting antioxidant defense and reducing ROS production.

### TRPM2 in innate inflammation and bacterial infection

Numerous studies have established the critical role of ROS/Ca^2+^ signaling in the immune system, covering aspects from immune cell activation to suppression of immune responses (Vig and Kinet, 2009; Nathan and Cunningham-Bussel, 2013; Yang et al., 2013; Morris et al., 2022). TRPM2, positioned at the intersection of ROS and Ca^2+^ systems, has quickly emerged as a target for investigating immune physiology (Knowles et al., 2013; Syed Mortadza et al., 2015). Early evidence from Perraud’s group and others provide indicates that TRPM2 is functionally expressed in inflammatory cells, including monocytes, neutrophils, and phagocytes (Perraud et al., 2001; Sano et al., 2001; Heiner et al., 2003; Massullo et al., 2006). Yamamato and colleagues showed that silencing TRPM2 prevents the H_2_O_2_-induced Ca^2+^ influx and the downstream Ca^2+^-dependent tyrosine kinase Pyk2. This, in turn, leads to NF-KB nuclear translocation and CXCL8 chemo-cytokine production in human U937 cells. These observations were further confirmed *in vivo* using TRPM2 TRPM2-KO mice. Notably, TRPM2-KO monocytes exhibited reduced CXCL2 production and exacerbated dextran sulfate sodium (DSS)-induced ulcerative colitis. However, TRPM2 KO neutrophil maintained their functions, including migration and infiltration from bone marrow in response to chemokines, suggesting that the protective role of TRPM2 inhibition may be primarily attributed to a defect in monocyte cytokine production (Yamamoto et al., 2008).

Using the carrageenan-induced inflammatory pain model, Haraguchi and coworkers showed that TRPM2-mediated inflammation critically depends on macrophage and microglial CXCL2 production and subsequent neutrophil infiltration (Haraguchi et al., 2012). Interestingly, TRPM2 deficiency had no effect on the production of H_2_O_2_, TNF-α and CCL2 involved in inflammatory pain (Ren and Dubner, 2010), suggesting that TRPM2 inhibition specifically reduces the inflammatory response. Another study also reported the involvement of TRPM2-mediated Ca^2+^ influx in lipopolysaccharide (LPS)-induced production of numerous cytokines (IL-6, IL-8, IL-10, and TNF-α) in primary monocyte THP-1 cells (Wehrhahn et al., 2010). Similarly, Yonezawa and collaborators demonstrated that inhibiting TRPM2 reduces bleomycin (BLM)-induced lung inflammation through decreased polymorphonuclear leukocyte (PMN) recruitment and reduced secretion of inflammatory cytokines, including IL-1β, MIP-2, and TNF-α (Yonezawa et al., 2016). In support, Malik’s group demonstrated that TRPM2-mediated Ca^2+^ influx is required for VE-cadherin phosphorylation at Y73, a process necessary for the disassembly of adherens junctions and opening of the paracellular pathways, thereby allowing PMN adhesion and transmigration (Mittal et al., 2017). Indeed, when challenged with LPS injection, TRPM2-depleted mice exhibited reduced PMN accumulation, inflammation, and mortality, suggesting a protective role of TRPM2 inhibition (Mittal et al., 2017). Using the 8-Br-cADPR TRPM2 antagonist, Eraslan and colleagues also showed the therapeutic benefits of TRPM2 inhibition, as it prevented ischemic acute kidney inflammation through reduced production of TNF-α, IL-1β, and myeloperoxidase activity (Eraslan et al., 2019). Moreover, suppression of TRPM2 alleviated fibrosis accumulation and the associated inflammatory response in a unilateral urethral obstruction (UUO) mice model, primarily by reducing transforming growth factor (TGF)-β1-mediated JNK activation and decreasing NF-kB expression and downstream fibrotic genes, including α-smooth muscle actin (α-SMA), connective tissue growth factor (CTGF), fibronectin (FN), and Collagen 1alpha 1 (Col1α1) (Wang et al., 2019). In accordance, inhibition of TRPM2 protected Apoe−/− mice from high-fat diet (HFD)-induced atherosclerosis by suppressing of CD36 signaling and the activation of macrophage pro-inflammatory activity (Zong et al., 2022c), further consolidating the benefits of TRPM2 inhibition in mitigating severe inflammatory responses.

In striking contrast, other studies have associated the loss of TRPM2 with the aggravation of inflammatory responses. For instance, Di and collaborators showed exacerbated proinflammatory cytokine production of CXCL2, TNFα, and IL-6 in the lungs and poor survival of TRPM2-deficient mice compared to WT mice in response to endotoxin LPS (Nathan and Cunningham-Bussel, 2013). Mechanistically, the authors showed that LPS activates TRPM2-mediated Ca^2+^/Na^+^ influx, which depolarizes the plasma membrane to inhibit NADPH oxidase and ROS production, proposing a negative feedback mechanism in which TRPM2 activation buffers LPS-induced ROS and proinflammatory activity (Wu and Tymianski, 2018). In another study, the inhibition of TRPM2 was associated with LPS-induced inflammation, neutrophil migration, and neutrophil-mediated vascular injury through a mechanism independent of TRPM2 cation channel activity (Wang et al., 2016a). The authors showed that LPS-induced ROS in neutrophils negatively regulates neutrophil migration by oxidizing TRPM2 at Cys549 of its N-terminal region (Wang et al., 2016a), which triggers interaction and internalization of the chemoattractant receptor FPR1, a key regulator of the inflammatory environment (Dorward et al., 2015). This finding suggests that TRPM2 activation may serve as a strategy mitigate neutrophil-induced inflammatory injury (Wang et al., 2016a).

In addition to its contribution to innate immunity, TRPM2 has also been shown to play a crucial role in defense against infections. For example, Knowles and colleagues reported that TRPM2-depleted mice were more susceptible to infection with *Listeria* monocytogenes (LM) and exhibited lower production of the cytokines IL -12 and IFNγ compared with WT mice, suggesting a role for TRPM2 in combating LM infections (Knowles et al., 2011). Partida-Sanchez’s group further showed that the increased susceptibility of TRPM2-KO mice to LM-induced lethality was associated with enhanced neutrophil migration, accumulation, and inflammatory TNF-α, IL -6, and IL -10 production in the liver and spleen. Interestingly, the authors demonstrated that *in vivo* depletion of neutrophils in TRPM2 KO mice prevented LM -mediated systemic inflammation, confirming the benefit of TRPM2 activation in neutrophils for combating LM dissemination and preventing neutrophil-mediated tissue damage during *Listeria* infection (Robledo-Avila et al., 2020). Using *H. pylori* as an infection model, the same group showed that the loss of TRPM2 resulted in increased macrophage-mediated inflammatory gastritis and decreased *H. pylori* colonization compared to WT mice (Beceiro et al., 2017). In this context, the authors showed that *H. pylori* activates TRPM2-mediated Ca^2+^ influx, which in turn reduces NADPH oxidase-mediated ROS production and macrophage inflammatory activities. In TRPM2-depleted macrophages, *H. pylori* induces excessive Ca^2+^ overload and enhanced MAPK and NADPH oxidase activities, exacerbating macrophage production of inflammatory cytokines, suggesting that TRPM2 activation may represent a promising therapeutic strategy to control macrophage-mediated excessive inflammation and tissue damage in response to *H. pylori* (Beceiro et al., 2017). Similar enhanced inflammatory activity has also been observed in TRPM2 KO mice in response to other infection models, including lung infection triggered by *P. aeruginosa* (Di et al., 2017), sepsis triggered by *E. coli* (Zhang et al., 2017), or polymicrobial sepsis (Qian et al., 2014).

The role of TRPM2 in the immune system is indeed multifaceted, as it has been shown to exert both proinflammatory and anti-inflammatory functions. The discrepancies between various reports may be attributed to factors such as the genetic background of the animal models employed, the specific context of inflammation under study, and the different pathways and doses of endotoxin-mediated inflammation. Consequently, additional research is required to evaluate the therapeutic potential of manipulating TRPM2 more accurately is innate inflammation and infection. This further investigation will facilitate a deeper understanding of TRPM2’s role and inform the development of targeted therapies for a range of inflammatory and infectious conditions.

## TRPM2 in cancer

Cancer survival has long been known to depend on its sustained metabolic rate, necessary for progression, and its antioxidant system, crucial for optimizing the production of reactive oxygen species (ROS). Simultaneously, growing evidence suggests that impaired Ca^2+^ homeostasis drives cancer development and progression. Being at the intersection of oxidative stress and Ca^2+^ systems, TRPM2 has rapidly become a fertile area of investigation for understanding cancer biology and improving cancer therapy. In fact, increased TRPM2 expression has been observed in numerous cancers, and its inhibition has been linked to cancer cell death *in vitro* and *in vivo*, implying its role in cancer survival and progression (Miller, 2019). In the following section, we highlight current knowledge regarding TRPM2-mediated Ca^2+^ entry in cancer signaling, from its connection to mitochondrial bioenergetics and redox balance to autophagy and chemotherapy response.

### TRPM2 in the mitochondrial bioenergetics and redox balance

In addition to providing energy in the form of ATP, mitochondria produce ROS from various sources, including the electron transport chain (ETC), mainly complexes I and III (Murphy, 2009; Zorov et al., 2014). Among the many factors that contribute to mitochondrial function and ATP production, Ca^2+^ plays an essential role coordinating oxidative phosphorylation and activating the respiratory chain, leading to increased ATP production (Brookes et al., 2004). In cancer, mitochondrial function is further stimulated to increase ATP production, which is required for sustained cell proliferation and survival, thereby also increasing ROS production and the risk of cytotoxicity (Sabharwal and Schumacker, 2014). In response, cells enhance antioxidant proteins to detoxify themselves from elevated ROS and cytotoxicity. In this context, Miller’s group have demonstrated a delicate role for TRPM2-mediated Ca^2+^ entry in mitochondrial bioenergetics and redox balance (Miller, 2019). In neuroblastoma cells, Miller’s group showed that inhibition or silencing of TRPM2 decreased hypoxia-inducible factor-1/2 (HIF)-1/2 and its downstream mitochondrial membrane proteins involved in mitophagy (BNIP3), ROS-scavenging (SOD1/2), and ATP synthesis (ETC., complexes) (Chen et al., 2014; Bao et al., 2016). In addition, downregulation of TRPM2 was associated with inhibition of Pyk2 and the downstream MCU, reducing mitochondrial Ca^2+^ uptake and altering the mitochondrial membrane potential (Hirschler-Laszkiewicz et al., 2018). Loss of TRPM2-mediated altered mitochondrial membrane proteins was also linked to altered mitochondrial function, as it resulted in significant reductions in oxygen consumption rate (OCR) and OCR-mediated ATP production, thereby suppressing energy production and ultimately reducing tumor survival and progression *in vitro* and *in vivo* (Chen et al., 2014; Bao et al., 2016; Hirschler-Laszkiewicz et al., 2018). Importantly, in rescue experiments using WT TRPM2 but not the silent E960D TRPM2 mutant, mitochondrial function and tumor survival were restored *in vitro* and *in vivo*, suggesting a direct link between TRPM2-mediated Ca^2+^ influx, mitochondrial bioenergetics, and cancer progression (Bao et al., 2016; Hirschler-Laszkiewicz et al., 2018). Similar observations have been made in acute myeloid leukemia (AML) (Xu et al., 2022), gastric cancer (Almasi et al., 2018), and breast cancer (unpublished data, El Hiani’s laboratory). Taken together, these data indicate that TRPM2 promotes cancer progression by enhancing mitochondrial function and ATP production. To mitigate the potential ROS accumulation associated with increased ATP production, cancer cells upregulate their antioxidant system, a key process in which TRPM2 has been shown to play a pivotal role. Indeed, Miller’s group demonstrated a critical role for TRPM2-mediated Ca^2+^ influx in maintaining the activity of Nuclear factor (erythroid-derived 2)-Related Factor-2 (Nrf2) (Bao et al., 2019), a master transcription factor that regulates crucial genes important for antioxidant enzymes and cofactors necessary to reduce excessive ROS levels in tumor cells (Abdul-Aziz et al., 2015; Tonelli et al., 2018). In neuroblastoma, the authors showed that loss of TRPM2 reduced the expression of Nrf2 and expression of its regulatory proteins IQ motif containing GTPase activating protein 1 (IQGAP1) and Kelch-like ECH-associated protein 1 (Keap1), as well as the expression of Nrf2 downstream antioxidant proteins NADP^+^, NADPH, NAD^+^, NADH, and GSH, leading to ROS accumulation. Moreover, ectopic expression of WT -TRPM2, but not the pore-silent TRPM2 E960D mutant, in TRPM2-depleted neuroblastoma cells restored cell viability and reconstituted expression of the antioxidants NADP^+^, NADPH, NAD ^+^, NADH, and GSH, while reducing ROS production, demonstrating the critical role of TRPM2-mediated Ca^2+^ entry in Nrf2 activity and downstream antioxidant proteins (Bao et al., 2019).

**FIGURE 2:**
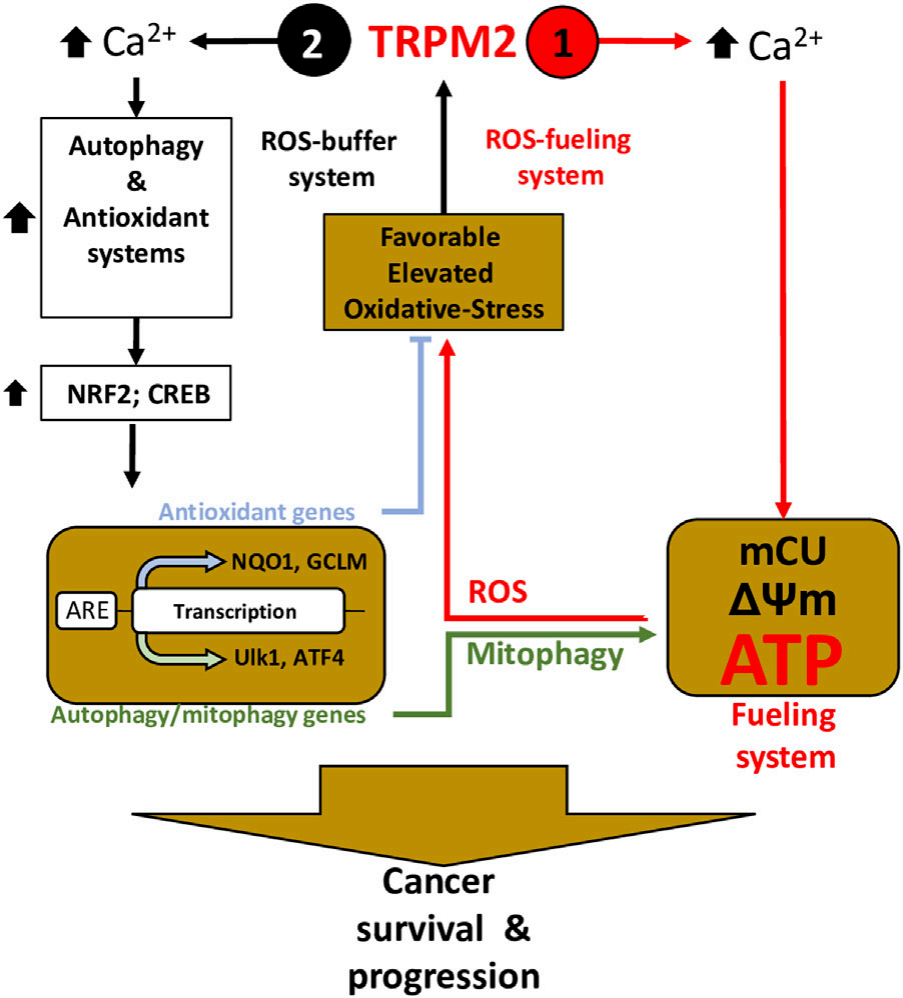
TRPM2 is essential in cancer survival and progression through the control of the fueling and oxidant-defense systems—Inspired from (Chen et al., 2020). Elevated ROS induces TRPM2-mediated Ca^2+^ to (1) directly coupled to mitochondrial Ca^2+^ uptake (mCU) to supply ATP production, thereby fueling program, and (2) trigger oxidant-defense by activating NRF2 phosphorylation, allowing its transfer and accumulation into the nucleus, which leads to the induction of a wide array of antioxidant genes. Simultaneously, TRPM2-mediated Ca^2+^ influx activates autophagy/mitophagy through the activation of CREB, ATF4, and ULK, thereby promoting mitophagy and maintenance of mitochondrial quality. On one hand, elevated ROS production guarantees increasing TRPM2 activity and thus increased ATP production, using a positive-feedback mechanism (1– ROS-On system); on the other hand, ROS-scavenging proteins and mitophagy buffer ROS from reaching levels that incur cell-damage (2—ROS-OFF system).

Collectively, these studies have shown that TRPM2 supports cancer survival through a dual function: Elevated cancer ROS activates TRPM2-mediated Ca^2+^ influx to 1) directly couple with mitochondrial Ca^2+^ uptake and ATP production to promote cancer progression, and 2) potentiate the NFR2 and downstream antioxidant proteins NADPH and GSH to reduce ROS production. On the one hand, increased ROS production ensures the enhancement of TRPM2 activity and thus increased ATP production via a positive feedback mechanism; on the other hand, TRPM2-mediated Ca^2+^ entry triggers ROS-scavenging proteins that prevent ROS from reaching cell-damaging levels (Figure 2).

### TRPM2 in autophagy and response to chemotherapeutics

Another specific adaptive system that cancer cells use to withstand elevated ROS -mediated cell death is autophagy. Autophagy plays an important role in controlling ROS levels (by eliminating dysfunctional proteins, recycling damaged organelles) and even in the induction of drug resistance (by removing DNA damage and eliminating mutant cells), thereby providing a survival advantage for cancer cell progression (Kumar et al., 2015; Chang and Zou, 2020; Redza-Dutordoir and Averill-Bates, 2021). However, there is also growing evidence that sustained autophagy may act as a suppressor of tumor development through excessive elimination of essential proteins and organelles, and the induction of caspase-independent autophagic cell death (Eskelinen, 2011; Singh et al., 2018; Yun et al., 2020; Zhou et al., 2020). Mechanistically, autophagy can be activated under various stress conditions, including nutrient starvation and hypoxia, and involves divers signalling pathways, particularly mTOR (mechanistic target of rapamycin) and ULK1 (unc-51-like kinase) (Dikic and Elazar, 2018; Yu et al., 2018). Since Ca^2+^ and ROS have been implicated in autophagic signalling pathways (Bootman et al., 2018; Hasan et al., 2022), many groups have investigated the role of TRPM2 in the autophagy of different types of cancer.

In this context, Miller’s group was the first to report a key role of TRPM2 in the link between autophagy and tumor growth (Chen et al., 2014; Bao et al., 2016). In neuroblastoma, this group demonstrated that inhibition of TRPM2 triggers cell death and is associated with a decrease in HIF-1/2 and its downstream target BNIP3, leading to the accumulation of Hsp60 and Tom20 proteins, two key indicators of decreased autophagy and mitophagy (Chen et al., 2014). Genetic deletion of TRPM2 in these cells also led to a decrease in HIF-1/2 and BNIP3, confirming the role of TRPM2 in autophagy (Bao et al., 2016). In support, Almasi and coworkers showed that silencing TRPM2-mediated cell death was associated with decreased autophagy flux and autophagy markers (e.g., ATGs 3, 5, 7, and 12) through an mTOR-independent JNK-dependent mechanism (Almasi et al., 2018). In acute myeloid leukemia (AML), Chen and coworkers also show that cell death mediated by knocking down TRPM2 is associated with decreased autophagy through inhibition of the CREB and ATF4, which are important transcription factors for autophagosome biogenesis, leading to a decrease in ULK1 and autophagy markers (ATGs 5, 7, and 13) (Chen et al., 2020). In striking contrast, Tektemur and coworkers showed that silencing TRPM2 induces apoptotic genes along with key autophagic genes, including ATG5, BECN1, and ULK1/2, suggesting a correlation between TRPM2 inhibition and autophagy induction (Tektemur et al., 2019). Using the TRPM2 antagonists CLT or 8-Br-ADPR, Wang and coworkers also showed that oxidative stress-mediated death of HeLa cells is accompanied by a TRPM2-dependent Ca^2+^ influx that activates CAMK2 (Ca^2+^/calmodulin-dependent protein kinase II) and its downstream target BECN1/Beclin 1, which in turn binds BCL2 to inhibit autophagy, making the cells more prone to death (Wang et al., 2016b). Collectively, these findings indicates that TRPM2 plays a pivotal role in cancer survival through a dual role in autophagy, which varies depending on the specific type of cancer.

In addition to its role in cell survival, numerous studies have shown that autophagy is involved in the efficacy of various chemotherapeutic agents and the development of drug resistance. While the mechanisms underlying autophagy’s impact on the effectiveness of anticancer drugs are not fully understood, a potential explanation could reside in the link between autophagy and ROS. Indeed, chemotherapeutic agents act primarily by inducing ROS to kill cells (Yang et al., 2018; Perillo et al., 2020). However, as mentioned above, TRPM2-mediated NRF2 and autophagy decrease ROS levels and increase the ROS-induced damage threshold, thereby buffering the cytotoxicity of anticancer drugs. This reduction in the efficacy of chemotherapeutic agents contributes to drug resistance. Targeting the TRPM2 function could, therefore, represent a tipping point that can be exploited for improved cancer therapy (Table 1). Accordingly, numerous studies have shown that inhibition of TRPM2 increased the efficacy of anticancer drugs. In neuroblastoma, Miller’s group demonstrated that inhibition of TRPM2 increased the cytotoxicity of doxorubicin (Chen et al., 2014; Bao et al., 2016; Hirschler-Laszkiewicz et al., 2018; Bao et al., 2019; Hirschler-Laszkiewicz et al., 2022), a widely used anticancer drug in chemotherapy to treat various cancers (Minotti et al., 2004). A similar result was reported in myeloid leukemia, where knockdown of TRPM2 enhanced doxorubicin-mediated cell death (Chen et al., 2020). In breast cancer, inhibition of TRPM2 increased cell death after doxorubicin and tamoxifen (Koh et al., 2015), another commonly used chemotherapeutic agent in breast cancer therapy (Howell and Howell, 2023). In gastric cancer, inhibition of TRPM2 has been shown to increase the efficacy of doxorubicin and paclitaxel (Almasi et al., 2018), a widely used anticancer drug in cancer therapy (Mekhail and Markman, 2002). Overall, these studies suggest that inhibition of TRPM2 is a novel strategy to increase the efficacy of anticancer drugs and improve cancer therapy. However, other studies show the opposite, with the activation of TRPM2 increasing the efficacy of chemotherapeutic agents. Most of these studies were conducted by Naziroglu’s research group, showing that the activation of TRPM2 enhanced the anticancer drug-mediated ROS production and cell death in various types of cancers. For instance, curcumin-mediated TRPM2 activation enhanced ROS production and cell death in laryngeal squamous cancer cells (LSCC) after treatment with cisplatin (Gokce Kutuk et al., 2019), one of the most commonly used drugs for the treatment of squamous cell carcinoma (Pendleton and Grandis, 2013). Interestingly, paclitaxel-mediated ROS production and cell death in LSCC also involved TRPM2 activation (Kumbul and Naziroglu, 2022). In glioblastoma cells, the combination of paclitaxel with and resveratrol, a naturally occurring phytochemical that has been shown to sensitize multiple cell lines to numerous chemotherapeutic agents (Xiao et al., 2018), potentiated paclitaxel-mediated cytotoxicity and cell death through the activation of TRPM2 channel (Ozturk et al., 2019). The combination of cisplatin and Eicosapentaenoic acid, a component of omega-3 polyunsaturated fatty acids recently shown to influence the effects of many anticancer drugs (Ghoreishi et al., 2012; Bennouna et al., 2019), also enhanced cisplatin-mediated oxidant effects and cell death through TRPM2 activation in glioblastoma cells (Ocal and Naziroglu, 2022). Selenium-mediated TRPM2 activation also enhanced ROS production and cell death in glioblastoma cells after treatment with docetaxel (Ertilav et al., 2019), an important antineoplastic agent used in the management of multiple metastatic and non-resectable tumor types (Farha and Kasi, 2023). Similarly, honeybee venom melittin and silver nanoparticles enhanced cisplatin-mediated oxidant actions and cell death in glioblastoma cells through the activation of TRPM2 (Akyuva and Naziroglu, 2023; Ertilav and Naziroglu, 2023). Finally, the TRPM2 agonist cumene hydroperoxide (CMPx) increased the sensitivity of breast and colon cancer cells to 5-fluorouracil (5-FU) and leucovorin (LV), both individually and in combination (Guler and Ovey, 2018), both of which are widely used drugs in breast and digestive tract cancers (Machover, 1997; Sethy and Kundu, 2021). Collectively, these studies clearly suggest that activation of TRPM2 is a promising strategy to improve the efficacy of many anticancer drugs. However, the drugs used in these studies to activate and inhibit TRPM2 function are far from specific to TRPM2, and genetic knockdowns and/or overexpression are necessary to confirm this role of TRPM2 in pro-anticancer drug effects.

**TABLE 1:** Summary of chemotherapeutic antibodies combined with TRPM2 modulators.

Status of TRPM2	Anticancer agents	Study system	Effects on ROS levels and/or cell death	References
TRPM2 knockout by CRISPER	Doxorubicin	Neuroblastoma	Increased ROS and cell death	Chen et al. (2014); Bao et al. (2016); Hirschler-Laszkiewicz et al. (2018); Bao et al. (2019); Hirschler-Laszkiewicz et al. (2022)
TRPM2 knockout by CRISPER		AML	Increased ROS and cell death	Chen et al. (2020)
TRPM2 downregulation ShRNA		Gastric Cancer	Increased cell death	Almasi et al. (2018)
TRPM2 inhibition by 2-APB and ACA		Breast cancer	Increased cell death	Koh et al. (2015)
TRPM2 downregulation ShRNA	Paclitaxel	Gastric cancer	Increased cell death	Almasi et al. (2018)
TRPM2 inhibition by 2-APB and ACA	Tamoxifen	Breast cancer	Increased cell death	Koh et al. (2015)
Activation of TRPM2 by paclitaxel	Paclitaxel	LSCC	Increased ROS and cell death	Kumbul and Naziroglu (2022)
Potentiation of TRPM2 activity by resveratrol		Glioblastoma cells	Increased cell death	Ozturk et al. (2019)
Activation of TRPM2 by Curcumin	Cisplatin	LSCC	Increased ROS and cell death	Gokce Kutuk et al. (2019)
Potentiation of TRPM2 activity by Eicosapentaenoic acid		Glioblastoma cells	Increased cell death	Ocal and Naziroglu (2022)
Activation of TRPM2 by silver nanoparticle		Glioblastoma cells	Increased ROS and cell death	Akyuva and Naziroglu (2023)
Activation of TRPM2 by CMPx	5-FU and LV, both individually & in combination	Breast and colon cancers	Increased cell death	Guler and Ovey (2018)
Activation of TRPM2 by selenium	Docetaxel	Glioblastoma cells	Increased cell death	Ertilav et al. (2019)

## Conclusion and perspectives

The therapeutic potential of targeting TRPM2 in a variety of diseases is significant, largely due to its multifaceted role in cellular functions across a range of organs. This opens up the prospect for broad-spectrum therapies. For instance, inhibiting TRPM2 in the brain could potentially alleviate cognitive dysfunction in neurodegenerative disorders. On the other hand, the activation of TRPM2 in the heart might offer protective benefits against ischemia-reperfusion injury. Furthermore, modulating TRPM2 activities in immune responses and cancer could not only help to manage disease progression but also enhance the efficacy of existing treatments.

Despite this considerable promise, the clinical application of TRPM2 agonists and antagonists confronts several significant challenges. A primary hurdle is the dearth of selective and potent modulators capable of effectively targeting TRPM2 without impacting other cellular processes. For instance, 2-aminoethoxydiphenyl borate (2-APB) (Togashi et al., 2008), flufenamic acid (FFA) (Naziroglu et al., 2007), and the anti-fungal imidazoles clotrimazole and econazole (Hill et al., 2004), have been described as TRPM2 inhibitors. However, these substances are also known to impact other targets: 2-APB is a general inhibitor of most TRP channels (Bencze et al., 2015) and also activates IP3R (Huang et al., 2005); FFA inhibits a wide variety of TRP channels (Rae et al., 2012); clotrimazole inhibits Ca^2+^-activated K+ channels (Brugnara et al., 1995; Wu et al., 1999); and econazole inhibits TRPV5 and TRPV6 (Hughes et al., 2018). These off-target impacts underscore the urgency in identifying potent, TRPM2-specific drugs prior to their potential introduction into clinical trials. In this regard, the discovery of the non-inactivating TRPM2 variant by the Csanády group provides a valuable model for validating TRPM2 drugs, studying the mechanisms of drug binding in steady-state gating, and verifying drug specificity and sensitivity. Considering that TRPM2 is ubiquitously expressed in various tissues and cell types, including neurons, cardiac cells, and immune cells, a TRPM2 modulator must reach these different tissues in adequate concentrations to exert its therapeutic effects. However, the widespread distribution of TRPM2 also amplifies the risk of off-target effects and undesirable interactions, necessitating the development of delivery systems specifically tailored to target tissues where TRPM2 modulation is desired. The complexity of TRPM2’s function in disease-specific scenarios further muddies the waters in the design of effective therapeutic strategies. This makes striking the right balance between inhibition and activation of TRPM2 another significant challenge.

In conclusion, the manipulation of TRPM2 presents exciting therapeutic potential. However, realizing this potential will require extensive and careful investigation to 1) fully understand the mechanistic intricacies of TRPM2 activity and its downstream signaling, 2) the development of highly selective and potent modulators, 3) a strategic approach to biodistribution, and 4) the appropriate balance between inhibition and activation of TRPM2.
